# *CHEK2* **^∗^**1100delC Mutation and Risk of Prostate Cancer

**DOI:** 10.1155/2014/294575

**Published:** 2014-11-06

**Authors:** Victoria Hale, Maren Weischer, Jong Y. Park

**Affiliations:** ^1^Department of Cancer Epidemiology, Moffitt Cancer Center, 12902 Magnolia Drive, Tampa, FL 33612, USA; ^2^Department of Clinical Biochemistry, Herlev Hospital, 2730 Herlev, Denmark

## Abstract

Although the causes of prostate cancer are largely unknown, previous studies support the role of genetic factors in the development of prostate cancer. *CHEK2* plays a critical role in DNA replication by responding to double-stranded breaks. In this review, we provide an overview of the current knowledge of the role of a genetic variant, 1100delC, of *CHEK2* on prostate cancer risk and discuss the implication for potential translation of this knowledge into clinical practice. Currently, twelve articles that discussed *CHEK2* ^∗^1100delC and its association with prostate cancer were identified. Of the twelve prostate cancer studies, five studies had independent data to draw conclusive evidence from. The pooled results of OR and 95% CI were 1.98 (1.23–3.18) for unselected cases and 3.39 (1.78–6.47) for familial cases, indicating that *CHEK2* ^∗^1100delC mutation is associated with increased risk of prostate cancer. Screening for CHEK2^∗^1100delC should be considered in men with a familial history of prostate cancer.

## 1. Introduction

Prostate cancer is the most common nonskin malignancy among men worldwide. In the US, an estimated 233,000 new cases and 29,480 deaths are expected in 2014 [1]. Prostate cancer is nevertheless a little understood disease. Unlike other common cancers, environmental risk factors appear to be of minor consequence. The strongest risk factor is for the individual man with a family history of the disease. Indeed, twin studies have suggested that about 42% of the individual's risk of prostate cancer may be attributed to heritable factors [2]. Further, recent genome wide association studies (GWAS) have identified susceptibility variants [3–5], providing evidence in support of the role of genetic susceptibility in the development of prostate cancer.

The cell cycle checkpoint kinase 2 (*CHEK2*) encodes the CHEK2 enzyme, which plays a critical role in ensuring accurate DNA repair in response to double-stranded breaks. Located on chromosome 22q12.1 [6],* CHEK2* spans 50 kb [7] and contains 14 exons [6]. CHEK2 kinase is activated by ataxia telangiectasia mutated (ATM) and phosphorylates TP53 and BRCA1, which activates the homologous recombination repair pathway [8, 9]. Because of the role of CHEK2 kinase in activating the repair mechanism of double-stranded DNA breaks, CHEK2 kinase acts as a tumor suppressor promoting genomic stability and thereby preventing tumorigenesis [10, 11]. Therefore, any mutation causing a malfunction of the CHEK2 protein decreases cellular ability to protect the integrity of DNA. Among numerous mutations identified in* CHEK2, *the best studied mutations in prostate cancer are 1100delC, IVS2+1G>A, I157T, and del5395 [12–17]. Three mutations (1100delC, IVS2+1G>A, and del5395) cause* CHEK2* protein-truncation. A missense variant I157T affects the forkhead-associated (FHA) domain, where it mediates ATM-dependent CHEK2 phosphorylation and targeting of CHEK2 to bind partners such as BRCA1 [18] and is associated with reduced DNA repair ability and increased risk for various cancers [19].

We decided to focus on the 1100delC mutation as it is a frame-shift mutation that abrogates kinase activity and renders the protein ineffective. In the following, we provide an overview of the current knowledge of the role of a genetic variant, 1100delC, of* CHEK2* in prostate cancer risk along with a meta-analysis and discuss the implication for potential translation of this knowledge into clinical practice.

## 2. Methods

An online search in PubMed was performed in December 2013 using the following keywords: (CHEK2^∗^1100delC prostate), (CHEK2^∗^1100delC), (CHEK2 prostate), (CHEK2 cancer), or (susceptibility gene prostate). Respectively, the PubMed provided 9, 36, 98, 326, and 1,590 results. References of the identified studies were scrutinized in order to make sure all possible studies could be included in this review. The titles and abstracts of articles identified by PubMed were reviewed and unmatched articles which did not meet with selection criteria were excluded. We included articles that examined* CHEK2* ^∗^1100delC heterozygosity and risk of prostate cancer. Overall, we found 12 articles that met this criterion [20–31].

In case results from the same individuals were reported in more than one publication, the newest or most informative article was selected [25–31]. Among the seven articles published by Cybulski et al. [20, 25, 26, 30, 31, 69, 70], we considered the most recent case/control study [20] as the most informative study. Additionally, three studies from Wu et al. [27], Dong et al. [23], and Zheng et al. [28] seem to contain data from the same cohorts. Therefore, Dong et al. study [23] was taken into consideration for analysis. Thompson et al. reported nonsignificant (*P* = 0.26) risk increase among* CHEK2 *mutation carriers [29]. However, the population of this study was based on families with breast cancer history and incidence rates of prostate cancer were based on questionnaire. In addition, genotyping analysis was done with only a fraction of study participants (1,324/11,116). Thus, out of 12 articles, five articles contained independent results applicable to be analyzed (Table 1) [20–24]. The seven excluded articles for conclusion were listed in Table 2 [25–28, 30, 31, 70]. There are a few studies that investigated associations between* CHEK2* ^∗^del1100C and prostate cancer risk. However, the investigators combined multiple mutations into a group. Therefore, we could not evaluate a role of* CHEK2* ^∗^1100delC mutation in these articles [56, 69, 70].

## 3. Results

The five included studies examined CHEK2^∗^1100delC heterozygosity and risk of cancer in 6,228 prostate cancer cases including 830 familial cases and 9,258 male controls. Selected characteristics of studies are shown in Table 1 [20–24]. Overall,* CHEK2* ^∗^1100delC was identified in 0.7% (36/5,124) of sporadic prostate cancer cases, 1.2% (13/1084) of familial prostate cancer cases, and 0.36% (33/9,258) of male controls. The pooled results of OR and 95% CI were 1.98 (1.23–3.18) for unselected cases and 3.39 (1.78–6.47) for familial cases, showing that* CHEK2* ^∗^1100delC heterozygosity is associated with increased risk of prostate cancer.

Cybulski et al. sequenced the coding region of* CHEK2* using genomic DNA from 140 Polish prostate patients and investigated a role of three variants, including* CHEK2* ^∗^1100delC in prostate cancer risk. The numbers of* CHEK2* ^∗^1100delC carriers were 4, 3, and 1 in 1,921 controls, 690 unselected cases, and 98 familial cases, respectively. The publication concluded that prostate cancer risk was nonsignificantly increased 2.0- and 4.9-fold for unselected and familial cases [30]. This however may have been a question of insufficient statistical power. Gene variants of* CHEK2* have been associated with predisposition of multiple cancers. Therefore, Cybulski et al. [31] tested whether* CHEK2* is a multisite cancer susceptibility gene using 4,000 controls and 4,008 various cancer cases. For prostate cancer, the* CHEK2* ^∗^1100delC mutation increased a nonsignificant risk (OR = 1.74, 95% CI = 0.48–6.35) based on 690 prostate cases and 4,000 controls. Later, Cybulski et al. [25, 26] reported a significant risk increase for* CHEK2* ^∗^1100delC mutation in unselected (OR = 3.5, 95% CI = 1.6–7.5) and familial cases (OR = 5.6, 95% CI = 1.6–19.9) as compared with controls. Recently, Cybulski et al. (2013) further investigated that four variants of* CHEK2*, including 1100delC mutation on risk and progression using expanded 3,750 Polish unselected prostate cancer patients, 412 familial cases, and 3,956 controls. Strong associations were observed for both unselected (OR = 3.2, 95% CI = 1.4–7.5) and familial cases (OR = 5.5, 95% CI = 1.6–19.0) [20].

Mayo investigators [23, 27, 28] analyzed gene mutations of* CHEK2*. Thirty-three different mutations were identified in* CHEK2* from 876 DNA samples from various prostate cancer patients. Most* CHEK2* mutations identified in prostate cancer patients were not detected in 423 control men. Frequencies of* CHEK2* ^∗^1100delC mutation were 0.022 (4/178 prostate tissues), 0.0025 (1/400 cases without familial history), 0.003 (1/298 cases with family history), and 0.0 (0/423 control men). The authors also investigated the functional importance of* CHEK2* ^∗^1100delC mutation [23]. Western blot analysis of* CHEK2* ^∗^1100delC mutation in the EBV-transformed cell lines showed a significant reduction of CHEK2 protein, which is involved in the DNA damage pathway. Based on these results, the authors concluded that CHEK2 may contribute to prostate cancer risk because the DNA-damage-signaling pathway probably plays a significant role in prostate cancer development [23].

Seppälä et al. reported that the frequency of* CHEK2* ^∗^1100delC was elevated in sporadic cases (OR = 3.14; 95% CI = 0.65–15.16) and in 120 prostate cancer patients with family history (OR = 8.24; 95% CI = 1.49–45.5) compared to 480 controls. These data suggest strong association between the 1100delC mutation and prostate cancer risk, especially in individuals with family history. Based on these data, the authors concluded that the* CHEK2* ^∗^1100delC mutation is a low-penetrance prostate cancer predisposition allele that contributes significantly to familial clustering of prostate cancer at the population level [22].

Weischer et al. reported whether* CHEK2* ^∗^1100delC mutation affected prostate cancer risk in the Danish general population with a prospective study design. They reported that multifactorially adjusted hazard ratio by* CHEK2* ^∗^1100delC heterozygosity versus noncarriers was 2.3 (95% CI = 0.6–9.5) for prostate cancer [24].

However, Wagenius et al. could not confirm results from Cybulski study. They assessed the significance of the* CHEK2* ^∗^1100delC mutation for prostate cancer in the population of Southern Sweden. Frequency of the* CHEK2* ^∗^1100delC mutation was not different in sporadic cases (1/145, 0.007) and in prostate cancer patients with family history (4/254, 0.016) compared to controls (3/305, 0.01) [21].

Thompson et al. assessed the risk of various cancers in association with* CHEK2* ^∗^1100delC mutation by using incidence data from 11,116 individuals from 734 non-BRCA1/2 families with breast cancer history. These data were from the United Kingdom (236 families), the Netherlands (233 families), Germany (17 families), and the United States (248 families) [29]. Thompson et al. tested 1,324 individuals from 734 families for* CHEK2* ^∗^1100delC mutation and identified 115 carriers from 67 families. Based on these data, the authors estimated relative risk to carriers and noncarriers by maximum likelihood via the expectation-maximization algorithm. Seventy-five prostate cancers were observed. Among these patients, six patients were in* CHEK2*1100^∗^delC-positive families. The ration of carrier RR (1.42) to the noncarrier RR (0.53) was 2.68 (*P* = 0.26). This study has a limitation because of the heavy reliance on family members' reports of cancer in their relatives. Therefore, the extent of the underreporting for male relatives was hindered to obtain meaningful estimates of the risk to male carriers because families were collected for breast cancer research projects.

## 4. Discussion

We reviewed the current knowledge of the role of a genetic variant, 1100delC, of* CHEK2* in prostate cancer risk and found pooled odds ratios of prostate cancer for* CHEK2* ^∗^1100delC heterozygote of 1.98 (95% CI: 1.23–3.18) for unselected cases and 3.39 (95% CI: 1.78–6.47) for familial cases versus noncarriers, suggesting that screening for CHEK2^∗^1100delC should be considered in men with a familial history of prostate cancer.

The* CHEK2* ^∗^1100delC mutation was first identified in patients with Li-Fraumeni syndrome in 1999 [71]. This mutation was presented as a cause of breast cancer by Meijers-Heijboer et al. [42] and has emerged as a potential risk factor of prostate cancer by Dong et al. [23]. This association was supported by other studies [20–22, 24, 25]. The prevalence rate of* CHEK2* ^∗^1100delC mutation is about 0.5–1% in Northern and Eastern European population [72]. However, this rate is much less frequent in other European countries. Furthermore, the* CHEK2* ^∗^1100delC mutation is very rare or absent in Hispanics, Blacks, and Asian populations [35, 39, 45, 52, 73, 74]. Although the prevalence of the* CHEK2* ^∗^1100delC mutation varies, risk of prostate cancer was found to be doubled for White men of Northern or Eastern European descent with heterozygous genotype for the* CHEK2* ^∗^1100delC mutation.

Bahassi et al. demonstrated the biological role of* CHEK2* ^∗^1100delC* in vivo*. They showed an increased tumor formation in multiple cancer sites, including lung and breast in homozygous* CHEK2* ^∗^1100delC mutant (*P* = 0.025) and heterozygous mice (*P* = 0.13) as compared with wild-type [75].

### 4.1. Role of* CHEK2* Mutation in Other Cancers

A role of* CHEK2* ^∗^1100delC was also evaluated in various cancers, including breast [32, 76], colorectal, glioma, melanoma [24, 59, 77], esophageal, ovarian, pancreatic, leukemia, and lung cancers (Table 3). Generally,* CHEK2* ^∗^1100delC heterozygosity increases the risk for breast, colon, melanoma, and prostate cancers. However, larger studies are needed to evaluate a potential association with* CHEK2* ^∗^1100delC mutation in other cancers, such as glioma, and pancreas.

The* CHEK2* ^∗^1100delC has been most studied in breast cancer. Weischer et al. investigated the association of the* CHEK2* ^∗^1100delC mutant with risk for breast cancer (OR = 3.01, 95% CI = 2.53–3.58) using a meta-analysis with 22 independent studies containing 25,571 cases and 30,056 controls [32]. Based on these data, they suggested that this mutation should be screened for when testing for BRCA1/2 mutations. Overall, the majority of the studies found some degree of association between breast cancer and the* CHEK2* ^∗^1100delC mutant.

Xiang et al. [62] reported a significant association between* CHEK2* ^∗^1100delC and risk for colorectal cancer (CRC) in a meta-analysis with 6 studies [60]. The* CHEK2* ^∗^1100delC mutant was associated with sporadic CRC (OR = 2.80, 95% CI = 0.49–4.30), unselected CRC (OR 2.11, 95% CI = 1.41–3.16), and familial CRC (OR 2.80, 95% CI = 1.74–4.51).

Weischer et al. performed an association study on the Danish and German population while also conducting a meta-analysis with four different studies [24, 77]. The odds ratio for malignant melanoma for* CHEK2* ^∗^1100delC heterozygotes compared with noncarriers was 2.01 (95% CI = 1.03–3.91) in Danes, 1.41 (95% CI = 0.46–4.31) in Germans, and 1.71 (95% CI = 1.02–3.17) in both Danes and Germans. In the meta-analysis with 2,619 cases and the 17,481 controls, the authors found that there was an odds ratio of 1.81 (95% CI = 1.07–3.05) of malignant melanoma for* CHEK2* ^∗^1100delC heterozygotes in comparison to the noncarriers. The author concluded that* CHEK2* ^∗^1100delC heterozygotes have a twofold risk of malignant melanoma compared with noncarriers [59].

However, several studies reported inconsistent results. Three small studies were performed to see whether there was an association between* CHEK2* ^∗^1100delC and risk for glioma [68, 78, 79]. However, independent and combined data suggest that the* CHEK2* variant is not associated with glioma risk.

In an esophageal cancer study, Koppert et al. found 1.5% of 551 cases and 1.4% of 644 controls carry the* CHEK2* ^∗^1100delC mutation [63]. Similarly, there is no patient with* CHEK2* ^∗^1100delC mutation among 91 German head and neck cancer patients [64]. Therefore, the authors concluded that the* CHEK2* ^∗^1100delC mutation has no major contribution in carcinogenesis in the esophagus [63] and head and neck [64].

Only one study described* CHEK2* ^∗^1100delC and its association with ovarian cancer risk. Among 268 randomly recruited Russian ovarian cancer patients, two patients had the* CHEK2* ^∗^1100delC mutation, while one carrier was found in 821 controls. Thus, the author concluded that there is no significant association between* CHEK2* ^∗^1100delC and risk for ovarian cancer [65].

Sellick et al. found that there may be a low penetrance effect on risk of chronic lymphocytic leukemia (CLL) based on 973 cases and 1,620 UK controls. But there was no significant association found between* CHEK2* ^∗^1100delC and familial or sporadic leukemia (OR = 0.74, 95% CI = 0.32–1.7) [67].

In the lung cancer study, Huijts et al. could not confirm an association between* CHEK2* ^∗^1100delC mutation and lung cancer risk in 457 unrelated lung cancer patients [58].

Results from multiorgan cancer study by Cybulski et al. suggested an increased risk in thyroid and renal cancers although they were not in the significant level [31].

The studies performed in Malaysia [39], France [68], USA [54], Chile [53], Spain [52], Turkey [16], Malaysia [39], Czech [66], and Korea [45] did not find any individual with the* CHEK2* ^∗^1100delC allele. In many cases, sample sizes were often not large enough to detect a case with* CHEK2* ^∗^1100delC mutation. Further, as described, there are significant differences of prevalence of the* CHEK2* ^∗^1100delC mutation among different populations.

We are aware of some limitations of this meta-analysis. First, because of the lack of the individual level data of the reviewed studies, our reports were based on unadjusted published estimates; therefore, we were unable to adjust them by possible confounders such as age and other environmental risk factors. Second, the prevalence of* CHEK2* ^∗^1100delC was only sufficient in the European population but not in other ethnic groups. Therefore, the role of this mutation in other ethnic groups could not be assessed.

### 4.2. Future Perspective

The screening for the* CHEK2* ^∗^1100delC mutation was suggested for cancer prevention, especially for breast cancer [32, 80]. The rationale is that (1)* CHEK2* ^∗^1100delC was demonstrated numerously as a risk factor for various cancers, (2) the lifetime prostate cancer risk for men with* CHEK2* ^∗^1100delCmutation is 25–45%, and (3)* CHEK2* ^∗^1100delC analysis would be a single genotyping test with a low cost.

Because odds ratios were reported for prostate cancer with this mutation range between 2.0 and 3.0, it is reasonable to suggest screening for* CHEK2* mutations in men. Men with* CHEK2* mutations and family history of prostate cancer show a higher risk of prostate cancer. Therefore,* CHEK2* screening would be a useful strategy for prostate cancer among individuals with familial history. Unfortunately, the role of this mutation in survival or response to treatment in prostate cancer is not established yet. However, a recent study reported that breast cancer with* CHEK2* ^∗^1100delC is associated with a worse survival [81]. Thus,* CHEK2* could be a future target of cancer genetic test that could help in the detection and prevention of various cancers [82]. Genotyping for* CHEK2* ^∗^1100delC should be considered in men of Northern or Eastern European descent with a familial history of prostate cancer.

## Figures and Tables

**Table 1:** Characteristics and results of 5 prostate cancer studies.

Carrier/Total	Population	Country	OR/HR (95% CI)	*P* value	Ref.
21/3,750	Unselected cases	PL	3.2 (1.4–7.5)	0.009	[20]
4/412	Familial cases		5.5 (1.6–19.0)	0.01	
7/3,956	Controls		Reference		

1/145	Without family history	SW	0.70 (0.07–6.78)	0.76	[21]
4/254	Familial cases		1.61 (0.36–7.27)	0.53	
3/305	Controls		Reference		

7/537	Unselected cases	FI	3.14 (0.65–15.16)	0.15	[22]
4/120	Familial cases		8.24 (1.49–45.54)	0.02	
2/480	Controls		Reference		

4/178	Cases	US	NA	NA	[23]
1/400	Without family history		NA	NA	
1/298	Familial cases		NA	NA	
0/423	Controls		NA	NA	

2/114	Cases	DE	2.3 (0.6–9.5)	NA	[24]
21/4,094	Controls		Reference		

**36/5,124**	**Unselected cases**	**Pooled**	**1.98 (1.23–3.18)**	**0.004**	**Combined**
**13/1,084**	**Familial cases**		**3.39 (1.78–6.47)**	**0.0001**	
**33/9,258**	**Controls**		**Reference**		

PL: Poland, SW: Sweden, FI: Finland, US: United States, DE: Denmark, NA: not available.

**Table 2:** Characteristics and results of 7 excluded prostate cancer studies.

Carrier/Total	Population	Country	OR/HR (95% CI)	*P* value	Ref.
14/1,864	Unselected cases	PL	3.5 (1.6–7.5)	0.002	[25, 26]
3/249	Familial cases		5.6 (1.6–19.9)	0.02	
12/5,496	Controls		Reference		

3/84	Cases	US	NA	NA	[27, 28]

	Families with breast cancer history	UK, NE, DE, US	2.68 (ratio of carrier RR versus noncarrier RR)	0.26	[29]

3/690	Unselected cases	PL	2.1 (0.5–9.4)	0.32	[30]
1/98	Familial cases		4.9 (0.5–44.6)	0.11	
4/1,921	Controls		Reference		

3/690	Unselected cases	PL	1.74 (0.48–6.35)	0.39	[31]
10/4,000	Controls		Reference		

PL: Poland, UK: United Kingdom, NE: Netherlands, DE: Denmark, US: United States, NA: not available.

**Table 3:** Characteristics and results of various cancer studies.

Cancer Site	Case/control	Population	Country	OR/HR (95% CI)	*P* value	Ref.
Breast	(459) 25,571/(179) 30,056	(Meta-analysis)		3.01 (2.53–3.58)		[32]

Breast	1,828/7,030	(Cases/controls)	UK, FI, NE, GE, RU	6.43 (4.33–9.56)	<0.0001	[33]

Breast	(120) 2,554/(37) 3,267	(Familial cases)	NE	4.30 (2.97–6.25)	<0.0001	[34]

Breast	3,882/8,609	(Cases/controls)	CA	2.6 (1.1–5.8)	0.05	[35]

Breast	75/300	(Cases/controls)	SW	2.5	0.26	[36]

Breast	708 bilateral + 1,395 unilateral	(Cases only)	US, DE	1.8 (0.6–5.4)		[37]

Breast	71/1,692	(Cases/controls)	NE	4.1 (1.2–14.3)	0.05	[38]

Breast	(0) 668	(Cases only)	MAL			[39]

Breast	1,101/4,665	(Cases/controls)	DE	1.2 (0.7–2.1)		[24]
		(Breast)		3.2 (1.0–9.9)		
		(Colorectal)		1.6 (0.4–6.5)		

Breast	161/153		US	NS		[40]

Breast	2,311/496	(Female cases/controls)	CA	6.65 (2.37–18.68)		[41]
			US	0.12 (0.02–0.89)		

Breast	1,071/1,620		UK, NE, CA, US, GE	1.1% of controls versus 5.1% of cases	0.00000003	[42]

Breast	300/1,665		US	1% among cases versus 0.3% of controls	0.1	[43]

Breast	10,860/9,065	(Aggregate)	UK, NE, FI, GE, AU	2.34 (1.72–3.20)	0.0000001	[44]

Breast	(0) 493	(Cases only)	KO			[45]

Breast	903/1,016		IR	0.5% of cases versus 0.1% of controls; 5.65 (0.66–48.46)	0.09	[46]

Breast	1,479	(Cases only)	US	2.1 (1.0–4.3)	0.049	[47]

Breast	1,035/1,885	(Positive family history)	FI	2.27 (1.11–4.63)	0.021	[48]
		(Bilateral)		6 (1.87–20.32)	0.007	

Breast	300	(Cases only)	AU	0.6% of cases		[49]

Breast	237/331	(Cases/controls)	NE	11.4% of cases versus 2.8% of controls	0.001	[50]

Breast	302	(Cases only)	RU	3% of cases.		[51]

Breast	(0) 400/(0) 400		SP			[52]

Breast	(0) 196/(0) 1,024		CHL			[53]

Breast	(0) 102	(Familial cases)	US			[54]

Breast	507/513	(Cases/controls)	FR	1.14% in cases versus 0.29% in controls; 5.18	0.004	[55]

Breast	5,953	(Cases only)	PL	3.6 (2.1–6.2)	0.0001	[56]
		(Prostate)		4.4 (2.2–8.7)	0.0001	
		(Colon)		4.2 (2.4–7.8)	0.0001	

Breast	8,612	(Cases only)	UK			[57]

Breast, Colorectal	75	(Cases only)	SW	2.5%	0.26	[36]

Breast	(3) 1,434	(Cases only)	NE	3.4 (0.4–32.6)	0.3	[58]

Melanoma	(15) 1,889/(59) 12,801	(Combined)		1.79 (1.02–3.17)		[59]
			DE	2.01 (1.03–3.91)		
			GE	1.42 (0.46–4.31)		

Melanoma	(18) 2,619/(67) 17,481	(Meta-analysis)		1.81 (1.07–3.05)		[59]

Colorectal	(8) 818/(5) 760	(Unselected)	SW	1.49 (0.49–4.58)	0.48	[60]
	(2) 174/(5) 760	(Familial)		1.76 (0.34–9.13)	0.50	

Colorectal	369	(Cases only)	NE	4.2% HNPCC cases		[61]

Colorectal	4,194/10,010	(Meta-analysis)		2.11 (1.41–3.16)	0.0003	[62]
	1,050/3,784	(Familial)		2.80 (1.74–4.51)	<0.0001	
	652/2,115	(Sporadic)		1.45 (0.49–4.30)	0.50	

Colorectal	(0) 210/(0) 446	(Cases/controls)	TUK			[16]

Esophagus	(8) 551/(9) 644	(Cases/controls)	NE	1.04 (0.35–3.06)	0.94	[63]

Head & Neck	(0) 91	(Case only)	GE			[64]

Ovary	(2) 268/(1) 821	(Cases/controls)	RU	6.17 (0.56–68.3)	0.09	[65]

Pancreas	(0) 270/(0) 683	(Cases/controls)	CZ			[66]

Leukemia	(8) 973/(18) 1,620	(Cases/controls)	UK	0.74 (0.32–1.7)		[67]

Lung	(0) 457	(Cases only)	NE			[58]

Brain	(0) 79	(Familial cases)	FR			[68]

UK: United Kingdom, FI: Finland, NE: Netherlands, GE: Germany, RU: Russia, CA: Canada, SW: Sweden, US: United States, DE: Denmark, GE: Germany, MAL: Malaysia, AU: Australia, KO: Korea, IR: Ireland, SP: Spain, CHL: Chile, FR: France, PL: Poland, TUK: Turkey, and CZ: Czech.
